# Mutations in *ASXL1 *are associated with poor prognosis across the spectrum of malignant myeloid diseases

**DOI:** 10.1186/1756-8722-5-12

**Published:** 2012-03-21

**Authors:** Véronique Gelsi-Boyer, Mandy Brecqueville, Raynier Devillier, Anne Murati, Marie-Joelle Mozziconacci, Daniel Birnbaum

**Affiliations:** 1 Centre de Recherche en Cancérologie de Marseille; Laboratoire d'Oncologie Moléculaire; UMR1068 Inserm, Institut Paoli-Calmettes, Marseille, France; 2 Aix-Marseille Univ, Marseille, France; 3 Département de BioPathologie, Institut Paoli-Calmettes, Marseille, France; 4 Départements d'Oncologie Moléculaire et de Biopathologie, CRCM, Institut Paoli-Calmettes, UMR1068 Inserm, 27 Bd. Leï Roure, 13009 Marseille, France

**Keywords:** *ASXL1*, Gene mutations, Myeloid diseases

## Abstract

The *ASXL1 *gene is one of the most frequently mutated genes in malignant myeloid diseases. The ASXL1 protein belongs to protein complexes involved in the epigenetic regulation of gene expression. *ASXL1 *mutations are found in myeloproliferative neoplasms (MPN), myelodysplastic syndromes (MDS), chronic myelomonocytic leukemia (CMML) and acute myeloid leukemia (AML). They are generally associated with signs of aggressiveness and poor clinical outcome. Because of this, a systematic determination of *ASXL1 *mutational status in myeloid malignancies should help in prognosis assessment.

## 

Mutations in the *ASXL1 *(additional sex combs like 1) gene were first reported in 2009 in myelodysplastic syndromes [1]. *ASXL1 *maps to chromosome region 20q11, close to the *DNMT3B *gene, and belongs to a family of three paralogs. *ASXL1 *comprises 12 exons and is expressed in most hematopoietic cell types.

## Function of the ASXL1 protein

**Figure 1:**
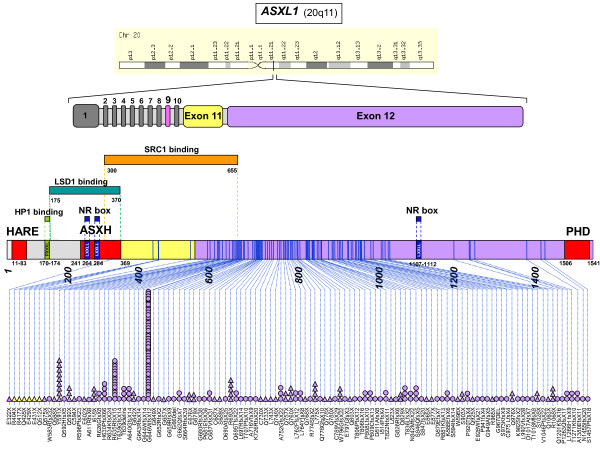
**Distribution of ASXL1 mutations along the protein**. From top to bottom are shown the localization of the *ASXL1 *gene on chromosome region 20q11, the exon structure of *ASXL1*, and the ASXL1 protein with its conserved motifs and binding regions: HARE helix-turn-helix at the N-terminus, HP1/CBX5 binding region, ASXH, an α-helical domain that contains LXXLL (nuclear receptor boxes), and the C-terminal plant homeodomain (PHD) finger. Below reported mutations (see Table 1) are shown along the protein: circles and triangles indicate frameshift and nonsense mutations, respectively, and the colors correspond to the exon location.

*ASXL1 *codes for a nuclear protein of 1084 residues characterized by an N-terminal helix-turn-helix domain, HARE-HTH [2], and an unusual C-terminal plant homeodomain (PHD), which may bind methylated lysines (Figure 1). The central part of ASXL1 contains an ASXH globular domain that may interact with a polycomb-associated deubiquitinase (DUB) [2,3]. ASXL1 regulates epigenetic marks and transcription through interaction with polycomb complex proteins and various transcription activators and repressors [3-5]. In *Drosophila*, ASX forms a complex with the ubiquitin carboxy-terminal hydrolase calypso to constitute the recently identified polycomb repressive deubiquitinase (PR-DUB) complex [3,6]. Human wild-type ASXL1 associates with the calypso ortholog BAP1 [7]. The calypso/BAP1 DUB deubiquitylates K119ub on histone H2A, leading to gene repression. However, the role of ASXL1 in leukemogenesis does not seem to be mediated by the DUB complex [7]. Recent data have shown that ASXL1 interacts with components of the polycomb complex PRC2, namely EZH2 and SUZ12, two proteins involved in the deposition of H3K27me3 histone repressive marks. These two PRC2 components are also mutated in myeloid malignant diseases [8-11]. Inhibition of ASXL1 function leads to loss of H3K27me3 histone marks. ASXL1 role could be to recruit the PRC2 complex to known leukemogenic loci such as *HOXA *genes [7]. ASXL1 also associates with HP1α/CBX5, a component of the heterochromatin repressive complex [6,12]. HP1α binds to histone H3. JAK2 phosphorylates histone H3 and excludes HP1α from chromatin [13]. Thus, a potential functional link may exist between *ASXL1 *and *JAK2 *mutations but this remains to be demonstrated.

The functions of the other ASXL proteins are poorly defined. ASXL2 has been shown to regulate heart [14] and bone development, as well as adipogenesis. Mouse ASXL2 has been identified as a regulator of bone mineral density and osteoclastogenesis [15] and whereas ASXL1 represses, ASXL2 increases the expression of adipogenic genes [16]. ASXL3 expression and functions remain to be determined [17].

## *ASXL1 *and concomitant mutations in myeloid malignancies

The vast majority of the *ASXL1 *mutations found in myeloid malignancies affect the twelfth exon of the gene although rare mutations in other exons have been detected [18]. *ASXL1 *mutations are frameshift and nonsense mutations that are supposed to result in C-terminal truncation of the protein upstream of the PHD finger (Figure 1). The functional relevance of some reported missense mutations is not clear. The most frequent mutation, which accounts for more than 50% of all *ASXL1 *mutations, is a duplication of a guanine nucleotide (c.1934dupG); it leads to a frameshift (p.Gly646TrpfsX12). One study has described this mutation as a PCR artefact [19], but because it is not found in germ-line DNAs, control DNAs or other studied types of cancers such as breast cancer, it is now generally considered to be a *bona fide *mutation.

*ASXL1 *mutations are usually heterozygous, suggesting that haplo-insufficiency is the key pathological factor; however, the truncated ASXL1 protein could also have a dominant negative role in titrating out an interacting protein. Actually, recent data have demonstrated a loss of ASXL1 protein in leukemia samples with ASXL1 mutation, indicating that these mutations are loss-of-function disease alleles [7].

*ASXL1 *is mutated in all types of malignant myeloid diseases, including myelodysplastic syndromes (MDS), myeloproliferative neoplasms (MPN), chronic myelomonocytic leukemia (CMML) and acute myeloid leukemia (AML). According to the series studied, *ASXL1 *mutation frequency varies from a few percent to more than 50% of cases (Table 1). *ASXL1 *mutations are most frequent in CMML (~ 45%). In MPNs, they are frequent in primary myelofibrosis (PMF)(34.5%) and rare in polycythemia vera (PV) or essential thrombocythemia (ET). In AML, they are found in secondary (30%) rather than in *de novo *cases (6.5%), and in AML with normal karyotype *ASXL1 *mutations are mutually exclusive with *NPM1 *mutations [20]. *ASXL1 *is the second most frequently mutated gene in MDSs after *TET2 *[21]. In MDSs, *ASXL1 *mutations are more frequent in refractory anemia with excess of blasts (RAEB) than in the other forms such as refractory anemia with ring sideroblasts (RARS) [1,5,22]. *ASXL1 *mutations are further detected in rare cases of juvenile myelomonocytic leukemia (JMML) [23] and in RARS-T [24].

**Table 1:** Mutations in *ASXL1 *gene in published studies

**Selected Ref**.	MDS n (%)	CMML n (%)	MPN n (%)	Secondary AML n (%)	*De novo *AML n (%)
*Abdel-Wahab et al., [25]				12/63 (19.3)	
*Abdel-Wahab et al., [18]		3/24 (12.5)	3/46 PMF (6.5)		
Béjar et al., [21]	63/439 (14.4)				
Brecqueville et al., [26]			17/149 (11.4): 6/30 PMF (20), 2/30 PV (7), 2/53 ET (4),		
Boultwood et al., [5]	28/182 (15.4)	17/51 (33.3)		9/40 (22.5)	8/27 (29.6)
Boultwood et al., [27]			+6/41 (CML) (14.6)		
Carbuccia et al., [28]			5/64 (7.8)		
Carbuccia et al., [20]				9/17 (58)	3/46 (6)
Chou et al., [29]					54/501 (10.8)
Gelsi-Boyer et al., [1]	4/35 (11.4)	17/39 (43.6)			
Gelsi-Boyer et al., [30]		25/53 (47.2)			
Grossmann et al., [31]		41/79 (52)			
Jankowsa et al.,[32]		24/52 (46)			
Pratcorona et al., [33]				3/24 (12.5)	35/775 (4.5)
Ricci et al.,[34]			23/42 PMF (54.8)		
Rocquain et al., [22]	13/65 (20)			9/18 (50)	3/46 (6.5)
Shen et al., [35]					27/605 (4.5)
Stein et al., [36]			12/47 PMF (25.5) 1/42 PV (2)		
Thol et al., [37]	40/193 (20.7)				
Total*	**148/914 (16.2)**	**124/274 (45)**	**41/119 PMF (34.5)**	**30/99 (30.3)**	**130/2000 (6.5)**

* not included in final count because p.Gly646TrpfsX12 had not been taken into account; + including CML cases

With the exception of *NPM1 *and *FLT3*, it seems that *ASXL1 *mutations coincide with mutations in many known genes including *EZH2 *[18], *IDH1/2, RUNX1 *and *TET2 *[21,22]. Although ASXL1 functions are related to the PRC2 complex, which includes EZH2, *ASXL1 *and *EZH2 *mutations are not mutually exclusive [18,38]. *ASXL1 *mutations can also cooperate with mutations in genes encoding signaling (*CBL, JAK2, NF1, RAS*) and splicing proteins (*SF3B1, SRSF2, U2AF35*). For example, in MDSs, *ASXL1 *mutations are more frequent in *U2AF35*-mutated patients than in *U2AF35 *wild-type patients [39]. In MPNs, *ASXL1 *mutations are found with the same frequency in JAK2V617F and JAK2 wild-type cases [26,36]. In MDSs, *ASXL1 *mutations are often associated with *RUNX1 *mutations, and, in AMLs, with *RUNX1 *and *CEBPA*. [29,33,40].

## Other alterations in *ASXL1, ASXL2 *and *ASXL3*

Few deletions of the gene have been reported and *ASXL1 *is generally not included in the more telomeric 20q13 deletion that is often observed in myeloid diseases. The *ASXL1 *gene can be translocated and fused to the *PAX5 *gene in acute lymphoblastoid leukemia [41] and altered by germ-line mutations in the Bohring-Opitz syndrome; this severe syndrome leads to death at an early age preventing to know whether susceptibility to hematopoietic diseases might result from *ASXL1 *germ-line mutations [42]. In recent genome sequencing studies rare mutations in *ASXL1 *and *ASXL3 *have also been found in chronic lymphocytic leukemia [43] but not in T-cell acute leukemia [44]. Mutations in *ASXL2 *and *ASXL3 *have not been found in myeloid diseases so far, but *ASXL2-MYST3 *and *EPC1-ASXL2 *fusions have been identified in myelodysplastic syndrome and T-cell acute leukemia, respectively [45,46]. Both MYST3 and EPC1 are epigenetic regulators and these fusion proteins probably disrupt epigenetic protein complexes.

## Animal models of ASXL1 loss

In a first model of *Asxl1 *gene knock-out in the mouse ASXL1 loss mildly perturbed myelopoiesis but did not trigger an actual hematological malignancy [47]. However, the effect of the absence of ASXL1 protein may have been masked by partially penetrant perinatal lethality. In another, more recent model of conditional *Asxl1 *gene knock-out, the animals developed a strong hematopoietic phenotype consistent with an MDS with myeloproliferative features. In cooperation with NRAS oncogenic mutation the absence of ASXL1 triggered an MDS/MPN. These observations were confirmed by experiments in hematopoietic cells using shRNA directed against *ASXL1*, which were highly coherent with the expected role of ASXL1 in leukemogenesis [7].

## *ASXL1 *mutations in disease evolution

Like *TET2 *mutations, *ASXL1 *mutations are found in chronic and acute stages of myeloid malignancies. In a study of MPNs, with the exception of a single patient who acquired both *ASXL1 *and *TET2 *mutations, all patients with *ASXL1 *mutation at leukemic transformation already had *ASXL1 *mutation at the chronic stage [25]. In a series of secondary AML with multilineage dysplasia we found that in cases resulting from a transformation of a known MDS the same *ASXL1 *mutation was present at both the chronic and acute stages (Devillier et al., submitted). These observations suggest that *ASXL1 *mutations may constitute early hits in leukemogenesis and precede other alterations such as *JAK2 *and *TET2 *mutations [24,25,28]. However, there is also evidence to suggest that the opposite is true in some cases. In MPNs, for example, the proportion of *ASXL1 *mutations is higher in post-PV myelofibrosis (MF) and post-ET MF than in PV and ET. This suggests that the *ASXL1 *mutation may follow a JAK2 mutation and could therefore help predict the risk of evolution from PV and ET to MF [26,36,48]. As such, *ASXL1 *mutations may play a crucial role in the pathogenesis of PMF, as well as in the molecular progression from the chronic phase of a previous PV or ET to MF. Finally, in MDSs and CMML, *ASXL1 *mutations seem to be present in chronic phases and precede transformation and in rare cases, *ASXL1 *mutations can be lost or acquired during relapse of *de novo *AML [29].

## *ASXL1 *mutations in disease outcome

A number of studies have linked *ASXL1 *mutations to the outcome of malignant myeloid diseases. In a study of MPNs based on the DIPSS-plus score [49] (Dynamic International Prognostic Scoring System for primary myelofibrosis), *ASXL1 *mutation tended to be associated with an aggressive disease and a poor overall survival [26]. In a large study of PMF patients *ASXL1 *mutations were associated with shorter overall survival [50]. In CMML, the presence of an *ASXL1 *mutation could help predict transformation to AML [30]. In MDSs, *ASXL1 *mutations are associated with a reduced time to progression in AML and constitute an independent prognostic marker [37]. Finally, a study of 18 genes in a large cohort of MDSs showed that mutations in 5 genes had prognostic impact: *TP53, EZH2, ETV6, RUNX1 *and *ASXL1 *[21]. Coupled with the standardized international prognostic scoring system (IPSS), mutations in these five genes could help refine the prognosis evaluation of MDSs.

By contrast, a study of a large cohort of 605 AML cases without cytogenetic prognostic markers other than 11q23 abnormalities, reported that *ASXL1 *mutations were not associated with outcome [35]. However, they were associated with shorter overall survival in patients with intermediate-risk AML [29,33]. A recent study of 476 cases with intermediate-risk *de novo *AML showed that *ASXL1 *mutations have a major impact on outcome [51]. According to the current European LeukemiaNet (ELN) guidelines for the diagnosis and management of AML, AMLs with normal karyotype are classified into two genetic categories based on their *NPM1, FLT3*-ITD and *CEBPA *mutation status: the ELN Favorable category is defined as mutated *CEBPA *and/or mutated *NPM1 *without *FLT3*-IT; all remaining cases (ie, those with wild-type *CEBPA*, and wild-type *NPM1 *with or without *FLT3*-ITD or mutated *NPM1 *with *FLT3*-ITD) form the ELN Intermediate-I category [52,53]. *ASXL1 *mutations have been associated with inferior survival among ELN Favorable, but not among ELN Intermediate-I patients [40]. Taken together, these data show that *ASXL1 *mutations have prognostic value in certain subgroups of AML patients.

## Conclusion

In almost all studies, and whatever the type of myeloid malignancy, *ASXL1 *mutations are associated with adverse features including, but not limited to myelodysplasia, myelofibrosis or progression to AML. Systematic detection of *ASXL1 *mutations could thus help in the assessment of disease and should perhaps be implemented in routine practice, whether associated with already systematically-surveyed mutations (*CEBPA, JAK2, FLT3, NPM1*) or in upcoming systematic genome analyses.

## Competing interests

The authors declare that they have no competing interests.

## Authors' contributions

All authors have contributed ideas, discussions, and have participated in the writing of the manuscript. All authors read and approved the final manuscript
